# Event and Comment

**Published:** 1906-01-15

**Authors:** 


THE
### DENTAL REGISTER.

Vol. LX. January 15, 1906. No. 1.

### EVENT AND COMMENT.

### Ohio State Dental Society.

The fortieth annual meeting of this society was held at Columbus, December 5th, 6th and 7th, and was one of the largest attended and most interesting meetings ever held. The meetings were held in the Great Southern Hotel, which afforded unusual accomodations for the exhibits, but rather poor accommodations for the meetings. Including the Presidents address and report of the State Examining Board there were ten papers presented and discussed. There were about sixty clinics given, some of which were very interesting and instructive. A large number of exhibitors were present and most of them had new appliances to show or demonstrate and to sell.

THE EXHIBITS.—It is surprising how interested most dentists are in new inventions, especially along the line of instruments and appliances. For the manufacturers and dealers and perhaps our patients too, it is a good omen to have dentists on the alert for new instruments and appliances which will expedite their work. If we could only afford to buy all these attractive appliances, there would be more pleasure in looking at them. It would seem that there never was a time when there was so much activity in the invention and marketing of new instruments and appliances. There were electric furnaces for baking porcelain inlays, crowns and bridges, two new ones, and several old timers; new furniture in the shape of cabinets and work benches; new fountain cuspidors; high pressure syringes for anesthetizing sensitive dentine; prophylaxis and other new instruments; porcelain teeth for crown and bridge work; and pulp destroyers, embalmers, disinfectants and antiseptics, more than you could carry away.

The Clinics.—These were unusually interesting and there were so many that it was difficult for anyone to get the benefit of all of them. The clinic room was not large enough to accomodate the clinicians and the audience, so that it was impossible for the clinicians to do their best work, especially as they had not only the crowd to contend with but the facilities for doing some clinics were not to be had. Dr. Ross who had charge of the arrangements, however, should be most cordially thanked for providing as many facilities that one would not expect in such a place. It would seem that such events should be held in the dental colleges where ample facilities for this kind of work are always available. The clinic as a feature of dental society work has developed wonderfully in the past two or three years. There are more men who seem willing to give clinics now than formerly, and those who attend seem disposed to place more value on the clinics than ever before. We like to see others do things because we can see how they overcome manipulative difficulties which have grieviously bothered us, or we sometimes find out that we can do something better than others and are correspondingly elated.

The Papers.—The first paper on "What to do with Erosions" was very generally discussed, and as is usual, there was no uniformity as to the cause or treatment. Of course the porcelain workers have no difficulty in treating this form of tooth destruction by porcelain restorations, and where the trouble is extensive this is probably the best form of treatment, but in some cases it is impracticable. The chemical treatment was advocated by several and objected to by others as a mere expedient which too often produces less desirable results than no treatment. The prophylaxis practitioners believe they have the only remedy where the conditions can be treated in the incipient stages. The thorough polishing of the affected areas and also of every surface of every tooth in the mouth results in producing such a normal condition of the gums that there are no vitiated secretions being constantly poured into the mouth to produce erosive results.

The report from the Board of Examiners was a statement of the work of the board for the year which has already been published. The report recommended that more legislation was necessary to make the administration of the law effective.

The teeth as essential or non-essential to civilized man was a characteristic paper by Dr. Wright and was undoubtedly inspired by a paper contributed to the American Medical Association last year by Dr. Fletcher. There seemed to be a general belief that teeth are necessary, to at least the civilized men, known as the dental profession, and that they are not only necessary but that they should be kept in as nearly their normal conditions as it is practicable to keep them by the practitioners of orthodontia, prophylaxis, operative and prosthetic dentistry and by any and all available means. When the predigested food people carry their propaganda to the extent of eliminating the function of the teeth there will be no occasion for discussing such a question, as the teeth will eliminate themselves by natural processes. The paper on "How to Treat Simple Cases of Orthodontia" came precious near demonstrating the fact that there are really very few simple cases of orthodontia, as one tooth with a tendency to wander from the path of duty will necessarily induce others to follow. The modern methods of treating even the simpler cases are so much more effective and satisfactory, that the profession is rapidly coming to realize the importance of early attention to these conditions. The beautiful results secured by early treatment are so manifest that it becomes a duty now for the conscientious practitioner to recommend to parents the correction of these conditions just as soon as they appear and not wait to see if they will not right themselves in time. The next three papers were read together as a symposium on prophylaxis. The interest in the subject as presented from its therapeutic, medical and technical aspects, was perhaps the feature of the meeting. The presentation of the medical aspect by a physician was remarkable for the unusual intelligence of the essayist on the medical relations of dental practice. The discussion of the subject showed by its spontaneous character that the profession is awakening to the fact that dentistry ought to be practiced as a profession and not as a trade. The discussion made clear that we shall need in the future to make our technical operations as much from the hygienic as the mechanical viewpoint.

The paper on the "Use and Abuse of Porcelain" was a very instructive paper and showed that its author had labored to great advantage in developing the scientific use of this, which is destined to be the ideal tooth filling material. It is to be regretted that no adequate discussion of the paper could be had, because of the lateness of the hour.

The lecture by Dr. G. V. Black on "Cavity Preparation" was one of the finest expositions of the rational and scientific treatment of this subject we have ever heard. The lecture was fully illustrated by lantern slides. Each class of cavity was taken up and described from the decayed tooth to the fully prepared and filled cavity in some cases. It was a splendid demonstration of the much misunderstood "Extension for Preventive Theory" of the author, and no one who heard that lecture and saw the illustrations will ever be led astray on that point again. It was so thoroughly rational that it almost seems that the last word has been said on that subject. It seems too bad that such a clear and wholesome statement of this subject cannot be incorporated in our text books on operative dentistry in place of the vagarious and indecisive methods which characterize them. The fundamental principle of cutting out decay and cavity form, as worked out by Br. Black, can be utilized in the manipulation of all materials and the emphasis, which he places on complete restoration of contour if accepted and practiced, will do more to save the reputation of the American Dentist than almost any other thing. Dr. Black could do no better service to his profession than to give this lecture before every dental society in the land.

